# Three dimensional imaging of gold-nanoparticles tagged samples using phase retrieval with two focus planes

**DOI:** 10.1038/srep15473

**Published:** 2015-10-26

**Authors:** Tali Ilovitsh, Asaf Ilovitsh, Aryeh Weiss, Rinat Meir, Zeev Zalevsky

**Affiliations:** 1 Faculty of Engineering, Bar Ilan University, Ramat-Gan 5290002, Israel; 2 The Bar-Ilan Institute of Nanotechnology & Advanced Materials, Bar Ilan University, Ramat-Gan 5290002, Israel

## Abstract

Optical sectioning microscopy can provide highly detailed three dimensional (3D) images of biological samples. However, it requires acquisition of many images per volume, and is therefore time consuming, and may not be suitable for live cell 3D imaging. We propose the use of the modified Gerchberg-Saxton phase retrieval algorithm to enable full 3D imaging of gold-particle tagged samples using only two images. The reconstructed field is free space propagated to all other focus planes using post processing, and the 2D z-stack is merged to create a 3D image of the sample with high fidelity. Because we propose to apply the phase retrieving on nano particles, the regular ambiguities typical to the Gerchberg-Saxton algorithm, are eliminated. The proposed concept is presented and validated both on simulated data as well as experimentally.

Light microscopy is the most popular technique used in biological imaging applications^1^,^2^, and is capable of providing 3D images of biological samples, which enables inspection of spatially resolved complex subcellular structures. The Rayleigh criterion^3^ defines the classical diffraction limit, which is the minimal distance at which two point sources can be resolved by conventional imaging systems. In an optical microscope, the lateral and the axial resolutions are ~200 nm and ~600 nm respectively. Objects below these dimensions appear as a radial diffractive ring pattern, which is the point spread function (PSF)^4^,^5^. The basic 3D imaging technique is optical sectioning, which requires scanning the sample at different depths in the longitudinal direction. The z-stack images are combined using post processing into a 3D image^6^. In order to obtain a high resolution 3D image, the z-steps between sequential frames must be small. Therefore, it is time consuming, and is not always practical for imaging of dynamic processes in living cells. Another category of 3D imaging includes digital holographic microscopy that is based on an interferometric setup between the imaging system and a reference beam. However, this requires the addition of a reference beam and an interferometric setup^7^,^8^.

Visualization of the interior of living cells using the visible-light microscope is difficult, due to the relative transparency of biological samples. Therefore, the samples are usually labeled with appropriate markers, in order to allow their visualization. The labeling is divided into two major categories. One utilizes fluorescent proteins (FPs) or other fluorescent probes as biomarkers^9^,^10^,^11^,^12^. The research in this area is extensive and includes super resolution techniques like (f)PALM^13^,^14^ and STORM^15^ and more evolved techniques^16^,^17^,^18^. However, typical drawbacks of fluorescence imaging methods are autofluorescence of live cells, the phototoxicity of FPs to living organisms and photobleaching^19^,^20^,^21^,^22^,^23^. An alternative choice is the use of gold nano particles (GNPs) as biomarkers^24^. GNPs exhibit the localized surface plasmon resonance (SPR) effect, which is manifested by enhanced absorption and scattering at a specific optical frequency when the optical illumination matches this resonant wavelength^25^. There are many studies on 2D imaging of samples using GNPs^24^,^26^, but 3D imaging using GNPs as biomarkers has very few published works. One technique uses photothermal optical lock-in optical coherence microscopy. However, this technique requires capturing both phase and amplitude images of the sample using a complex and expensive interferometric setup^27^,^28^. Phase retrieval algorithms can be used in order to reconstruct 3D objects with a set of captured images at two different focus planes. This concept was already demonstrated for 3D samples^29^. However, it required 9 images in order to perform the reconstruction and in addition it resulted mainly in the reconstruction of the exterior 3D shape, while lacking the ability to provide highly detailed mapping of the interior of the cells. Here, we propose the use of phase retrieval for 3D imaging of GNP-labeled samples. The GNPs can enter the cell, and can be targeted to specific areas within the cell, and thus serve as a selective contrast agent capable of providing high resolution imaging of the GNP-labeled elements within the cell. We suggest the use of the modified Gerchberg–Saxton (GS) algorithm^30^,^31^ for performing the phase retrieval task using only two images. There are a number of reports of phase reconstruction using two images. One is used for wavefront sensing in order to measure the aberrations of optical lenses, and is based erase duplicated phrase on a maximum likelihood algorithm^32^. This algorithm yields similar results to the GS algorithm, but it converges more slowly and is sensitive to aberrations. Another uses a modified GS algorithm in order to perform wavefront correction in a shaped-pupil coronagraph for the detection of extra-solar terrestrial planets^33^. Applied to their data sets, a conventional GS algorithm with only two images will not converge due to ambiguities associated with the algorithm. Therefore, they suggest placing a binary mask that passes light only in the dark hole region that they wish to correct in order to guarantee the convergence of the GS algorithm using two images. An ambiguous image is one whose FSP is identical to the FSP of a second image that is other than a scaled version, a translation, or a twin of the image. As a result, there are multiple solutions that the GS algorithm can converge to and this is referred to as the GS algorithm ambiguity. One way to overcome this is to capture a third plane image and apply the algorithm to three planes instead of only two^34^. Another way to eliminate this ambiguity steams from the fact that if the object has finite support (it is zero outside a finite region), it is considered to be unique and a single solution is guaranteed to exist for the GS algorithm^35^. The major advantage of our proposed technique is that the GNPs are single objects with a point-like structure and with finite support. Their choice as the objects to be imaged eliminates the ambiguity associated with multiple solutions to the GS algorithm and enables its rapid convergence to the correct solution with only two images and without additional components, making it attractive for real time 3D imaging applications. A necessary condition for this phase retrieval algorithm is spatially coherent illumination. Therefore, fluorescent proteins can’t serve as the contrast agents in the proposed technique, whereas GNPs are suitable.

## Theoretical Background

The 3D PSF of an aberration-free defocused imaging system with a finite lens aperture is given by an Airy function^36^ that expands with defocus. Each optical section includes both in-focus plane and out-of-focus contributions that are determined by the corresponding pupil function^37^. In the absence of aberrations, the standard deviations of the 3D PSF is given by^37^,^38^,^39^:


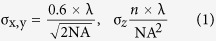


where NA is the numerical aperture of the objective, *λ* is the wavelength of the emitted light and *n* is the refractive index of the medium between the coverslip and the objective front lens element. A z-stack is a set of optical sections that describes the 3D object, and often includes tens to hundreds of images. However, by taking only two images and retrieving the phase and intensity of the propagating light, one can reconstruct the 3D object, as well as planes that were not imaged. In addition, because the algorithm is continuous, it can also be used to reconstruct planes that are between sequential planes (i.e. at any z-step *Δz*_*i*_).

**Figure 1:**
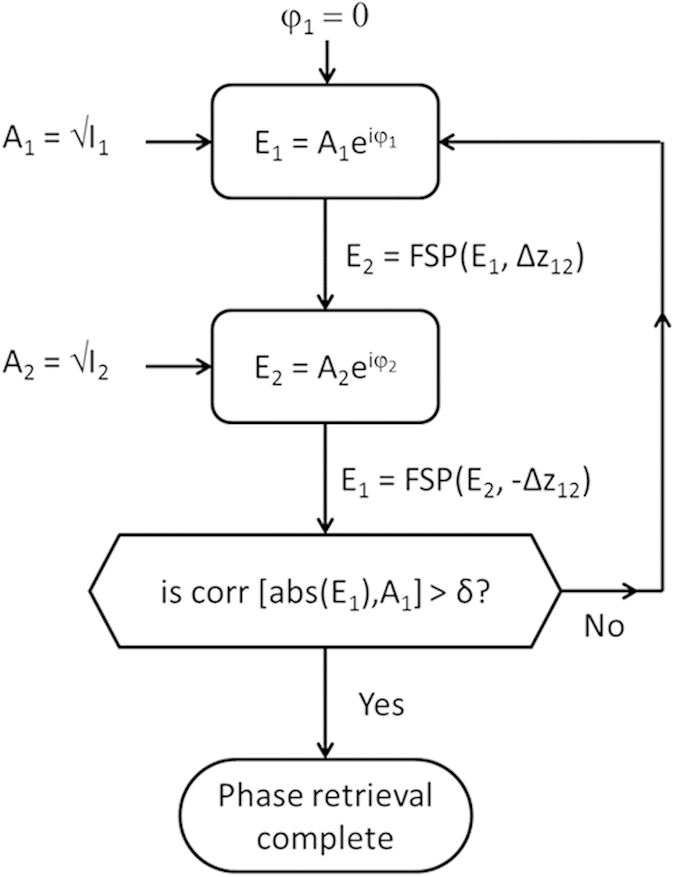
Flowchart of the GS process.

The phase retrieval is done using an iterative process that relies on the revised GS algorithm. The two captured intensity images are termed *I*_*i*_, where *i* = *1,2*, and their fields E_i_ = √I_i_. The field’s amplitude and phase are A_i_ and φ_i,_ respectively, and the distance between the planes is *Δz*_*i*_. The GS algorithm is implemented as shown schematically in Fig. 1. The amplitude A_1_ is initially inserted with an imposed zero phase φ_1_. This field undergoes free space propagation (FSP) for a distance of *Δz*_*12*_ to the second plane. The result is the amplitude and phase that define the second plane E_2_. The known amplitude of A_2_ is imposed, while the calculated phase φ_2_ is kept. The field E_2_ undergoes another FSP over a distance of −*Δz*_*12*_ to the input plane E_1_, where the known amplitude of A_1_ is imposed, while the calculated phase φ_1_ is retained. This process is iterated until the correlation coefficient between A_1_, calculated with FSP, and the known acquired amplitude is higher than a predetermined threshold. Once the phase retrieval process is completed, this known field can be FSP to any plane, including planes that were not captured during the imaging process.

## Simulations

**Figure 2:**
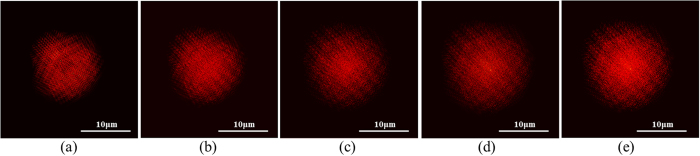
Simulation images. (**a**–**e**) A set of 5 sequential images at distances of 1 *μm* out of the simulation set. The images have been contrast-stretched and were pseudo-colored to make them visible in print.

The simulated model was of a 3D sample that contains GNPs, with a scattering peak at wavelength of *λ* = *540* *nm,* that was imaged through an objective lens onto a CCD camera and was scanned along the Z axis, resulting in a z-stack set of images of the sample. The model’s parameters were chosen to correspond to an optical system consisting of a *63x/NA* = *1.4* oil immersion objective lens, imaged though a 1.0x relay lens onto a CCD sensor array with *6.45* *μm* × *6.45* *μm pixels*, which translates to *102* *nm* × *102* *nm* in the object plane. The z-step size was *Δz*_*i*_ = *100* *nm* and the generated set contained *100* z-stack images. The value for σ_*x,y*_ and σ_*z*_ for the given imaging parameters, was calculated from Equation (1) to be *167* *nm* and *413* *nm* respectively. The simulated model was of a single cell, with a diameter of 10 *μm* and a thickness of 5 *μm*, that contained randomly positioned GNPs within the 3D simulated cell. 100 GNPs were inserted into each plane. Figure 2 is a set of *5* sequential images with a spacing of *1* *μm* from the simulation set. Each image contains in-focus and out of focus PSFs. The PSF shape expands with de-focus, thus widening the simulated cell’s shape as larger the Z offsets are from focus.

**Figure 3:**
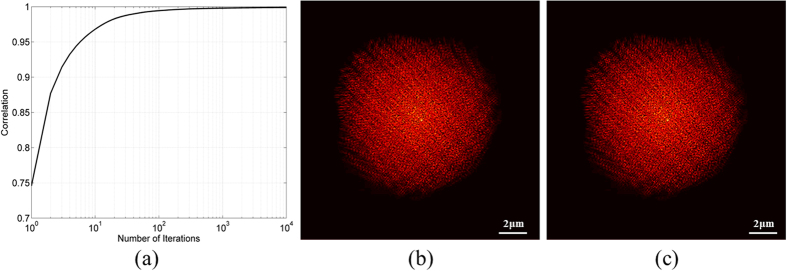
Simulation results. (**a**) The correlation graph between the reconstructed image and the original one as a function of the iteration number. (**b**) is the image that was generated by the FSP of the reconstructed field for a distance of *4* *μm* and (**c**) is the originally generated image at distance of *4* *μm*. Both images are presented with *40*% increased brightness and contrast and were pseudo-colored for their better visualization.

Two simulated images out of the 100 simulated z-stacks were chosen, and the GS algorithm was applied to them. The phase retrieval algorithm enabled recovery of each of the other simulated frames, as well as well as planes that weren’t imaged, by performing FSP of the obtained field using post processing. Figure 2(a,c) are the two images used for the GS algorithm. Figure 3(a) presents the correlation graph between the original captured images and the reconstructed one using the GS algorithm as a function of the iteration number. The correlation exceeds *90%* after only *5* iterations, and is almost 100% at *500* iterations. Figure 3(b) is the image that was generated by FSP of the reconstructed field for a distance of *4* *μm,* and Fig. 3(c) is the originally generated image at distance of *4* *μm*. The correlation coefficient between Fig. 3(b,c) is 99.998%.

**Figure 4:**
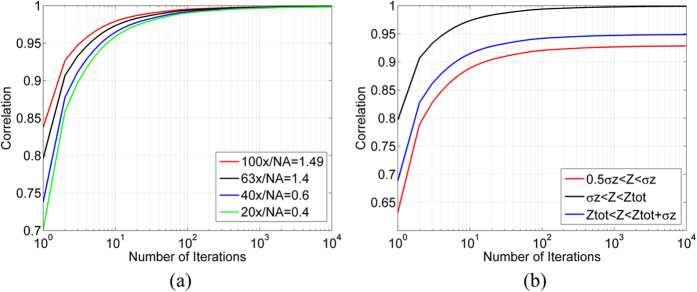
Correlation graphs. (**a**) Correlation graphs for different NAs and magnifications. (**b**) Correlation graphs as a function of the Z distance between the two images.

Figure 4(a) shows the correlation graphs for different NAs and magnifications. All of the correlation graphs converge to 1, but the number of iterations required to achieve this value changes as a function of the NA and magnification. Higher magnification produces a more detailed image, resulting in more rapid convergence of the GS algorithm.

Another important consideration is the Z distance between the two planes used for the GS algorithm. They should be chosen such that there is sufficient FSP between the two, so that each PSF will significantly change, thus providing sufficient data for the GS algorithm to converge. When the Z distance is smaller than 0.5 σ_*z*_, the algorithm will not converge and the method will not succeed. Figure 4(b) shows the correlation graphs as a function of the Z distance between the two images, where the minimal distance is 0.5 σ_*z*_ and the maximal is the thickness of the sample (Z_tot_)+ σ_*z*_ for 63x/NA = 1.4. The optimal choice is a Z distance that is between σ_*z*_ and the thickness of the sample. A Z distance that is between 0.5 σ_*z*_ and σ_*z*_ will converge more slowly and to a value of 93%, due to insufficient change in the PSF. This results in the regular ambiguities typical of the GS algorithm. When the distance is larger than the thickness of the sample, Z_tot_, the intensity of the captured image deteriorates rapidly and as a result, so does the performance of the GS algorithm until it ceases to converge at a distance greater than Z_tot_ + σ_*z*_ and the method is no longer applicable.

## Materials and Methods

### Materials

#### GNP Synthesis

GNPs were prepared using sodium citrate according to the known methodology described by Enustun and Turkevic^40^. 0.414 mL of 1.4 M HAuCl_4_ solution in 200 mL water was added to a 250 mL single-neck round bottom flask and stirred in an oil bath on a hot plate until boiled. 4.04 mL of a 10% sodium citrate solution (0.39 M sodium citrate tribasic dihydrate 98%, Sigma cas 6132-04-3) was then quickly added. The solution was stirred for 5min, and then the flask was removed from the hot oil and set aside until cooled.

### GNP Conjugation

In order to prevent aggregation and to stabilize the particles in physiological solutions, *O*-(2-Carboxyethyl)-*O*′-(2-mercaptoethyl) heptaethylene glycol (PEG7) (95%, Sigma-Aldrich, Israel Ltd.) was adsorbed onto the GNPs. This layer also provides the chemical groups required for conjugation (-COOH). First, the solution was centrifuged to remove excess citrate. PEG7 solution was then added to the GNP solution, stirred overnight and centrifuged in order to remove excess PEG. Stabilized GNPs were further coated with glucose, in order to increase cell-uptake rate. Excess EDC (N-ethyl-N -(3-dimethylaminopropyl) carbodiimide) and NHS (N-hydroxysuccinimide) (Thermo Fisher Scientific, Inc, Rockford, IL) were added to the solution, followed by addition of Glucose-2 (2GF)(D-(+)-Glucosamine hydrochloride, Sigma-Aldrich, Israel Ltd.). NHS and EDC form an active ester intermediate with the -COOH functional groups, which can then undergo an amidation reaction with the glucose –NH_2_ group.

### Cell loading with GNPs

A431 cells were cultured in 5 ml glucose-free DMEM medium containing 5% FCS, 0.5% Penicillin and 0.5% glutamine. Cells were centrifuged and a saline solution containing GNPs at concentration of 0.9% was added in excess. The concentration of the GNPs within the cells depends on the incubation time. For low concentration the cells were then incubated at 37 °C for 20 minutes, whereas for high concentration the incubation time was 1 hour. After incubation, the cells were centrifuged twice (7 minutes in 1000 rpm) to wash out unbound nanoparticles. GNP-labeled-cells were incubated in Formaldehyde solution at room temperature for fixation and were placed on a glass slide. The slides were then covered with a #1.5 glass coverslip and sealed with nail-polish.

### Methods

**Figure 5:**
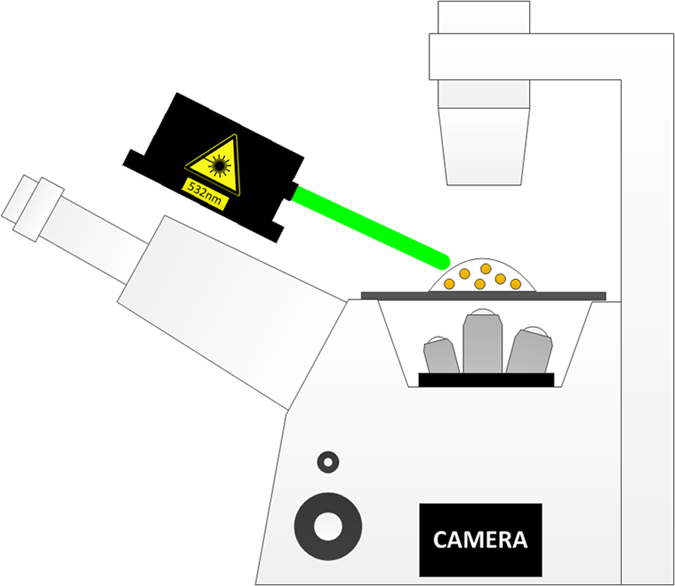
Experimental setup. A green laser at wavelength of 532 nm illuminates the sample and a z-stack set of images of the scattered light is collected.

Each sample was illuminated using a solid-state *532* *nm* laser (Photop DPGL-2100F) with a laser power of *8 mW*, mounted offset to the microscope stage, as shown in Fig. 5. The set of Z-stack images of the scattered light from the sample was acquired using a fully automated Nikon TE2000E inverted fluorescence widefield microscope, through a 40x/NA = 0.6 long working distance air objective (Nikon CFI S Plan Fluor ELWD 40X).

Images were acquired with a Retiga 2000R cooled CCD camera (QImaging, Surrey, BC, Canada) with *7.4* *μm* × *7.4* *μm* pixel size, thus the effective pixel size was *185* *nm*. The system was controlled with Nikon’s NIS Elements software. Multiple fields were acquired automatically, and in each field, a z-stack of *40* slices at *100* *nm* spacing was acquired at a frame rate of *7.5* *fps*. The multi-field ND2 images were preprocessed using the Fiji distribution of ImageJ^41^. The raw ND2 files were read using the Bioformats plugin^42^. The desired field and channel was extracted, and the image stack was cropped in order to reduce computation time. The cropped image stack was saved as a TIFF image sequence for further processing in MATLAB.

The GS algorithm was implemented in MATLAB (version 2012b, MathWorks, Natick, MA, USA). The program was run on a HP Compaq Elite 8300 Microtower PC with Windows 7 Professional 64 bit operation system, Intel® Core™ i5-3470 processor, 3.20 GHz, 12 GB RAM.

The camera’s frame rate is *7.5* *fps*, thus the acquisition of the 3D optical sectioning images was *6* seconds. The proposed method requires only two images, hence *0.2* seconds. The FSP of the reconstructed propagating field is done via post processing and thus is applicable for real time imaging purposes. There are available cameras that reach a frame rate at the order of *~100* *fps*, which accelerates the process even further to *20* *ms*.

## Experimental Results

**Figure 6:**
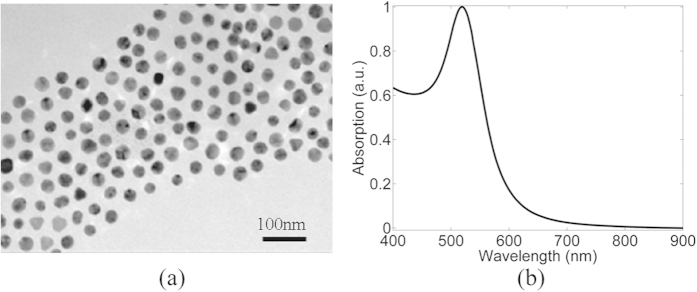
Characterization of GNPs. (**a**) TEM image of *20* *nm* GNPs. (**b**) NIR-visible spectroscopy of the GNPs. (Reprinted with permission from ref. [24]. Copyright NPG 2015).

A431 human epidermoid carcinoma cells^43^ were loaded with *20* *nm* spherical GNPs, as described above. GNP characteristics were measured using transmission electron microscopy (TEM), and the GNPs diameter was verified to be 20 *nm* (Fig. 6(a)). Their absorption spectrum was measured with a NanoDrop2000c spectrophotometer (Thermo-Scientific) and is shown in Fig. 6(b). In order to experimentally validate our approach, the method was applied to A431 cells loaded with GNPs. Two different concentrations of GNPs within the cells were used.

First, cells with low GNP concentration were used. The use of low concentration enabled the visualization of the defocus of the PSFs, which can be easily detected and provide a proof of concept of the proposed technique. Two images containing well focused GNPs were chosen out of the z-stack set, so that the defocussing of the PSF could be easily visualized. Using these two images, a third image was reconstructed, where different GNPs that were out of-focus in the original two images, came into focus. The reconstructed image was then compared to the image taken from the z-stack set, with the same z-step.

**Figure 7:**
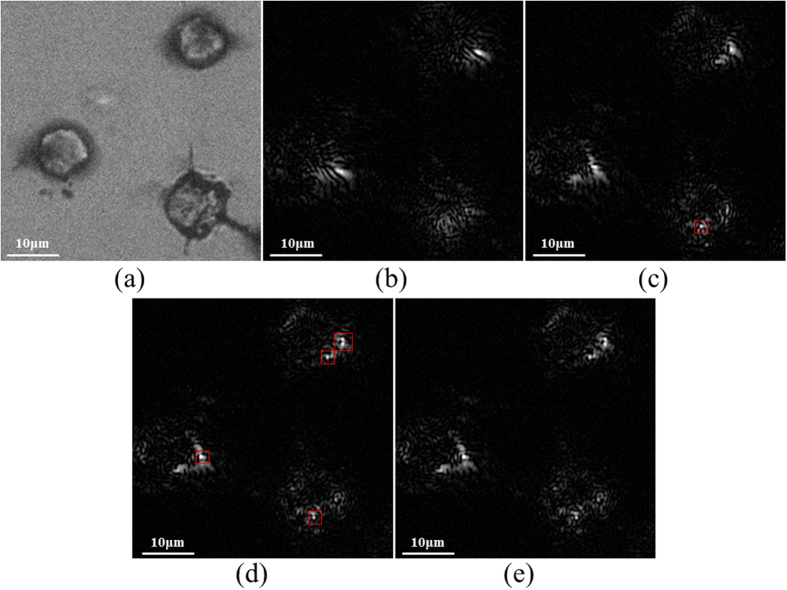
Experimental results. (**a**) a bright field image of the sample, which contains three cells. (**b**) and (**c**) are the two images, spaced by Δz = 2 μm, taken from the z-stack set and used for the GS algorithm. In-focus PSFs (chosen by the eye) are marked in red. (**d**), is a reconstructed image of a plane in the middle between the two images. (**e**) is the image from the z-stack set that was captured at the same plane as (**d**) and used for the authentication of the obtained results.

Figure 7(a) shows a cropped area of *300* × *300* pixels, taken from the bright field image, which contains three cells. The two images taken from the z-stack set and used for the GS algorithm are shown in Fig. 7(b,c), where in-focus GNPs (chosen by the eye) are marked in red. σ_z_ was calculated with Equation (1) to be *1.5* *μm*, and therefore the images were chosen such that they are spaced by *Δz* = *2μm*. Using these two images, a plane midway between the two images (*Δz* = *1μm* with respect to Fig. 7(b)) was reconstructed, and is shown in Fig. 7(d). In the area marked in red, there are a number of GNPs that come into focus, but were out of focus in the original two images. These results were validated by comparison to the corresponding experimental image acquired at the same *Δz* = *1μm*, shown in Fig. 7(e). The correlation coefficient between the images of Fig. 7(d,e) is 99.997%.

**Figure 8:**
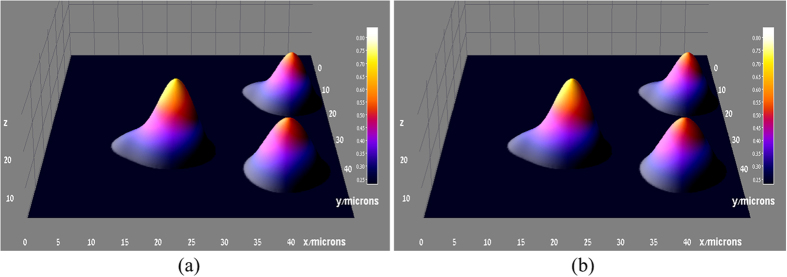
(**a**) The 3D reconstruction of the same sample used in Fig. 7. This reconstruction was done with the GS algorithm and the same two images (Fig. 7(b,c)). (**b**) The same 3D image generated using the original z-stack set.

**Figure 9:**
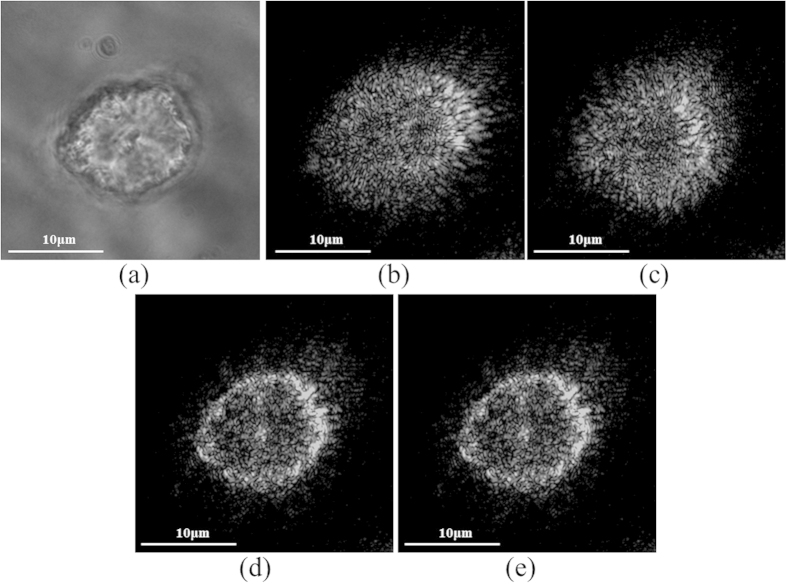
Experimental results with high GNPs concentration. (**a**) A phase microscopy image of a single cell within the sample. (**b**,**c**) are the two images, spaced by Δz = 4 μm, taken from the z-stack set and used for the GS algorithm. (**d**) is a reconstructed image of a plane in the middle between the two images. (**e**) is the image from the z-stack set that was captured at the same plane as (**d**) and used for the authentication of the obtained results.

The field which was calculated with the GS algorithm applied to the same two images (Fig. 7(b,c)), was then FSP to the other planes, resulting in a reconstructed series of the 2D z-stack images that were merged in order provide a 3D mapping of the cell (Fig. 8(a)). As the GNPs are evenly distributed within the cells, a well suited way to present the 3D combined data is to use a 3D surface plot. The same 3D image generated using the original z-stack set is presented in Fig. 8, the entire cell contains GNPs, which allows its visualization and reconstruction using post processing.The second sample was of high GNP concentration, which enables highly detailed mapping of the cell. Figure 9 (a) is a phase image of a single cell within the sample. This sample was imaged using the proposed approach. The same procedure previously described was applied to two images taken from the Z-stack set (Fig. 9(b,c)), that are spaced by *Δz* = *4μm*. Using these two images, a plane midway between the two images (*Δz* = *2μm* with respect to Fig. 9(b)) was reconstructed, and is shown in Fig. 9(d). The corresponding experimental image acquired at the same *Δz* = *2μm* is shown in Fig. 9(e), and the similarity to the reconstructed image validates the proposed approach. In addition, the captured images have a visible resemblance to the simulated model presented in Fig. 3.

## Summary and Conclusions

This paper presents a technique for 3D imaging of GNP-loaded cells using only two images. The proposed approach is based on the GS phase retrieval algorithm, and is generic and applicable to all wavelengths, given GNPs with an absorption peak that matches the laser’s wavelength. Scattered light from the GNPs is spatially coherent, which is a necessary condition for the GS algorithm. The method enables full 3D imaging of a sample with only two images, thus reducing the acquisition time necessary for imaging the 3D sample. In addition, the method can yield a specific volume mapping by targeting the GNPs into a specific volume.

## Additional Information

**How to cite this article**: Ilovitsh, T. *et al.* Three dimensional imaging of gold-nanoparticles tagged samples using phase retrieval with two focus planes. *Sci. Rep.* **5**, 15473; doi: 10.1038/srep15473 (2015).
